# The Study of Yak Colostrum Nutritional Content Based on Foodomics

**DOI:** 10.3390/foods12081707

**Published:** 2023-04-19

**Authors:** Lin Xiong, Jie Pei, Pengjia Bao, Xingdong Wang, Shaoke Guo, Mengli Cao, Yandong Kang, Ping Yan, Xian Guo

**Affiliations:** 1 Animal Science Department, Lanzhou Institute of Husbandry and Pharmaceutical Sciences, Chinese Academy of Agricultural Sciences, Lanzhou 730050, Chinapingyanlz@163.com (P.Y.); 2 Key Laboratory of Animal Genetics and Breeding on Tibetan Plateau, Ministry of Agriculture and Rural Affairs, Lanzhou 730050, China; 3 Key Laboratory of Yak Breeding Engineering in Gansu Province, Lanzhou 730050, China

**Keywords:** yak, colostrum, foodomics, functional ingredient, regulatory mechanism

## Abstract

The utilization of yak milk is still in a primary stage, and the nutrition composition of yak colostrum is not systematically characterized at present. In this study, the lipids, fatty acids, amino acids and their derivatives, metabolites in yak colostrum, and mature milk were detected by the non−targeted lipidomics based on (ultra high performance liquid chromatography tandem quadrupole mass spectrometer) UHPLC−MS, the targeted metabolome based on gas chromatography−mass spectrometer (GC−MS), the targeted metabolome analysis based on UHPLC−MS, and the non-targeted metabolome based on ultra high performance liquid chromatography tandem quadrupole time of flight mass spectrometer (UHPLC−TOF−MS), respectively. Meanwhile, the nutrition composition of yak colostrum was compared with the data of cow mature milk in the literatures. The results showed that the nutritive value of yak colostrum was higher by contrast with yak and cow mature milk from the perspective of the fatty acid composition and the content of Σpolyunsaturated fatty acids (PUFAs), Σn−3PUFAs; the content of essential amino acid (EAA) and the ratio of EAA/total amino acid (TAA) in yak colostrum were higher than the value in yak mature milk; and the content of functional active lipids including phosphatidylcholines (PC), phosphatidylglycerol (PG), phosphatidylserine (PS), lyso-phosphatidylcholine (LPC), lyso-phosphatidylglycerol (LPG), lyso-phosphatidylinositol (LPI), sphingomyelin (SM), ganglioside M3 (GM3), ganglioside T3 (GT3), and hexaglycosylceramide (Hex1Cer) in yak colostrum, was higher than the value of yak mature milk. Moreover, the differences of nutritive value between yak colostrum and mature milk were generated by the fat, amino acids and carbohydrate metabolism that were regulated by the ovarian hormone and referencesrenin-angiotensin-aldosterone system in yaks. These research results can provide a theoretical basis for the commercial product development of yak colostrum.

## 1. Introduction

In recent years, with the rapid development of society and the consumer’s concern on health, the public possesses a huge demand for nutritious and healthy food. The active factors of milk source are the basis of dairy deep processing, and also are the key of realizing the transformation of milk into functional raw materials. The exploration of functional factors in milk and the functional base materials in industrialization can promote the transformation and upgrading of dairy industry. By contrast with mature milk, the cow colostrum generated in the mammary glands during the first day after giving birth is rich in calories and protein [1]. Moreover, cow colostrum contains plenty of functional active substances such as immunoglobulins, oligosaccharides, polyunsaturated fatty acid (PUFA), antibacterial peptides, and growth factors [2], and plays an important role in nutrition metabolism, the growth and development of the gastrointestinal tract, and the endocrine function of calves. Studies show bovine colostrum possesses many biological activities to the human body, such as improving upper respiratory tract infections [3], ameliorating diarrhea [4], and enhancing immunity [5]. Therefore, cow colostrum can be developed and utilized as a kind of healthy functional food resource, and the American Food Science and Technology Association listed it as the non−herbal natural health food with the best development prospect in the 21st century. The application of cow colostrum was earlier developed and there are many products, such as the infant formulas, children snacks, cheese, protein bars, and solid drinks. The related functions mainly focus on immune and intestinal health, and it is worth mentioning that bovine colostrum has been used in oral beauty and daily chemical products.

Yak (*Bos grunniens*) is a unique, rare cattle in the Tibetan Plateau, and it can survive in the extremely harsh environment, such as low temperature, oxygen deficit, and strong ultraviolet [6]. It is considered as one of the most outstanding domestic animals in the world and provides local herdsmen with animal-derived food, fur products, and animal power. The lactation period of yak is short, whereas yak milk possesses the characteristics of high protein, fat and dry matter, plenty of functional fatty acids, while it is devoid of pollutants due to the unique growing environment [7,8]. Yak milk has been accepted by more and more people in recent years and is not only welcomed by Chinese people outside the Tibetan Plateau region but also many foreign consumers. As a kind of unique resource with high nutritional value, yak milk can fill the gap of high-end dairy products with the trend of functional food in the world. However, the development and utilization of yak colostrum are still in a primary stage due to the traditional production mode and insufficient attention to yak production. Meanwhile, there are few studies on yak colostrum at present, and a systematic study of the nutritional ingredient in yak colostrum has not been carried out. Therefore, it is necessary to clarify the active components and nutritional factors in yak colostrum to evaluate the nutritional value of yak colostrum.

Due to the complex composition of milk, which is easily affected by many factors, the traditional chemical analyses for milk nutrition are being confronted with great challenge. Foodomics is a comprehensive and high-throughput technology for the food science based on the improvement of human nutrition [9]. It is the combination of omics technologies including genomics, transcriptomics, proteomics, and metabolomics, and it can detect some exogenous compounds affecting human health. At present, this technology has been used in the researches of fermented food, genetically modified food, nutrition, and safety testing [10]. Lipidomics is a systems biology approach in life sciences and investigates the lipid alteration, and it has been successfully applied to studying the milk fat characterization of cows [11], human [12] and goats [13]. As a tool of foodomics, metabolome including non-targeted and targeted metabolite analysis mainly studies the small molecule metabolites produced by organisms, and it possesses great potential for studying the dynamic changes of food components. The milk metabolomic of humans [14], cows [15] and sheep [16] has been reported and plenty of functional active ingredients were found.

In this study, a relatively comprehensive study was carried out to observe the ingredient differences of yak colostrum and mature milk, and the specific nutritive value of yak colostrum and the formation mechanism was explored by foodomics. The fatty acids, amino acids and their derivatives, lipids in yak colostrum, and mature milk were detected by the targeted metabolome based on gas chromatography-mass spectrometer (GC−MS), the targeted metabolome based on ultra high performance liquid chromatography tandem quadrupole mass spectrometer (UHPLC−MS), and the non-targeted lipidomics based on UHPLC−MS, respectively. The specific active ingredients in yak colostrum were excavated. Further, the differential metabolites (DMs) in yak colostrum and mature milk were identified by the non-targeted metabolome based on the ultra high performance liquid chromatography tandem quadrupole time of flight mass spectrometer (UHPLC−TOF−MS) and then was annotated by the Kyoto Encyclopedia of Genes and Genome (KEGG) analysis. The generation mechanism of specific ingredients in yak colostrum was expounded. This study establishes a theoretical basis for the development of functional active ingredients in yak colostrum and promotes the high quality development of the yak milk industry.

## 2. Materials and Methods

### 2.1. Animals and Sample Collection

The experimental location was in the Gannan region, Gansu province, China, and the geographical coordinate lies on 100°46′−104°44′ E and 33°06′−36°10′ N. Six healthy female yaks (Kecai yak, 165.33 ± 15.18 kg, firstborn) were selected in this study. Because yak is a kind of large livestock and is grazed on pasture, it is very difficult to carry out this study in a large sample size for experimental animals. Therefore, six yaks were enough to finish this study, and they possessed a higher cost performance. The experimental yaks were dewormed before the test. All yaks were grazed in natural grassland and could freely eat grass and drink water. The grassland is the subalpine meadows, and the vegetation is dominated by *Cyperaceae* and *Gramineae*. After female yaks calved in May, the colostrum and mature milk were collected on the first day, two months later, respectively. An approximately 50 mL milk sample of each yak was put into a sterile tube and kept in an ice cooling box and then was quickly sent back to laboratory. In the laboratory, the milk samples were stored in an ultra-low temperature freezer (Haier-Biomedical, Qingdao, China) at −80 °C.

### 2.2. Determination of Fatty Acids with Targeted Metabolism Based on Gas Chromatography−Mass Spectrometer (GC−MS)

The content of fatty acids in yak milk was detected according to the method described in references [17,18] with some modifications. Five mL milk was centrifuged at 1500 r/min for 15 min, and then the upper−middle liquid was taken into a tube with plugs. Methanol and potassium hydroxide were added into the tube and then the solution was shaken for 2 h. The pH of solution was adjusted to 3 using hydrochloric acid. Ten mL n-hexane was added, and the tube was on standing for 10 min after shaking. The supernatant was transferred into a new tube and dried under nitrogen. Two mL 1% sulfuric acid-methanol solution was added into the tube. The mixture was put on an 80 °C water bath, and the process of methyl ester lasted for half an hour. Fatty acid methyl esters (FAMEs) were extracted with 2 mL n−hexane. Two mL saturated salt solution was added into the solution, followed by shaking and centrifugation at 3500 r/min for 2 min, and then the supernatant was transferred into a new tube. The solution was dehydrated using anhydrous sodium sulfate, and then centrifuged at 3500 r/min for 2 min. The supernatant was transferred into a new tube, and 25 μL methyl nonadecanoate was added as the internal standard, and then the mixture was dried under nitrogen. The residue was redissolved in 1 mL n−hexane, and the solution was filtered into a vial for analysis.

The prepared samples were detected with a 7890/5975 GC−MS (Agilent Corp., Santa Clara, CA, USA) coupled with DB-WAX capillary column (30 m × 0.25 mm ID, 0.25 mm) (Agilent Corp., Santa Clara, CA, USA). The temperature ramp program was as follows: the initial temperature was maintained at 50 °C for 3 min and then increased to 220 °C at 10 °C/min and held for 5 min. The injection volume was 1 μL, and the value of split/splitless was 1:100. The carrier gas was helium gas, and the flow rate was 1.0 mL/min. Inlet temperature was 280 °C. MS parameters were as follows: ion source temperature 230 °C, transmission line temperature 250 °C, electron impact ion (EI) source, signal ion monitoring (SIM) scanning mode, electron energy 70 eV. Quality control (QC) samples were set in the sample queue to evaluate the stability and repeatability of the detecting system. The mixed standard solution was prepared using the standard substance containing 40 FAMEs. The peak area and retention time of the chromatogram map were extracted by MSD ChemStation software. The standard curve was drawn and then the content of fatty acids was calculated by external standard method (Table S1). The linear relationship of fatty acids in the linear range was good, and the correlation coefficient (r) was no less than 0.9900. The limit of detections (LODs) was in 0.8−1.5 mg/L, and the limit of quantifications (LOQs) was in 1.5−4.8 mg/L. The recoveries were in 93.5−101.2%, and the relative standard deviations (RSDs) were in 2.2–3.7%.

### 2.3. Determination of Amino Acids and Their Derivatives with Targeted Metabolism Based on Liquid Chromatography Tandem Quadrupole Mass Spectrometer (UHPLC−MS)

The content of amino acids and their derivatives in yak milk was detected according to the method described in reference [19] with some modifications. A 5 mL milk sample was put into a hydrolytic tube and was diluted into 10 mL using the solution of 6 mol/L hydrochloric acid, and then 3 drops of phenol were added in the tube. The hydrolyzed tube was frozen for 5 min in an ice bath and then was filled with nitrogen. The sealed hydrolytic tube was placed in an electric blast thermostat for 22 h at 110 °C. The hydrolytic solution was filtered into a 50 mL volumetric bottle, and the hydrolytic tube was rinsed with a small amount of water. The solution in the volumetric bottle was diluted to 50 mL with water. One mL solution was transferred into a test tube and then the mixed solution was dried under nitrogen at 40 °C. The residue was redissolved in the moving phase, and the solution was filtered into a vial for analysis.

The prepared samples were separated with a 1290 Infinity LC system (Agilent Corp., Santa Clara, CA, USA) coupled with the Zic−HILIC column (2.1 mm × 150 mm, 3.5 µm) (Merck, Darmstadt, Germany). The column temperature, flow rate, and injection volume were 40 °C, 250 μL/min and 1 mL, respectively. The mobile phase consisted of A (25 mmol/L the solution of ammonium formate in water containing 0.08% formic acid) and B (acetonitrile containing 0.1% formic acid). The initial mobile phase was 90% B and then linearly decreased to 70% B in 12 min, 70–50% B over 12–18 min, 50–40% B over 18–25 min, 40–90% B over 30.1–37.0 min, holding at 90% B. The QC samples were used to evaluate the stability and repeatability of the detecting system. The MS data were collected with a 5500 QTRAP MS (AB SCIEX Corp., Framingham, MA, USA). The ESI source conditions were as follows: source temperature 500 °C, gas1 40, gas2 40, curtain gas 30, ISVF 5500 V. Multiple reaction monitoring (MRM) mode was used to detect the ion pairs. The peak area and retention time were extracted by Multiquant software. The content of amino acids and their derivatives was calculated by external standard method (Table S2). The linear relationship of targeted amino acids and their derivatives in the linear range was good, and the value of r was no less than 0.9900. The LODs were in 0.05–0.30 μmol/L, and the LOQs were in 0.18–0.80 μmol/L. The recoveries were in 88.5–103.0% and the RSDs were in 2.6–5.5% at low, middle, and high spiked levels.

### 2.4. Lipids Extraction and MS Data of Non−Targeted Lipidomics Based on UHPLC−MS

The lipids in yak milk were detected according to the method described in references [20,21] with some modifications. The lipids were extracted according to the methyl tertiary butyl ether (MTBE) method. Two hundred µL water was added into the milk sample, followed by vortexing for 5 s. Subsequently, 20 µL the solution of lyso PC17:0 in methanol (0.1 mg/mL) and 240 µL methanol were added, and the solution was vortexed for 30 s. After that, 800 µL MTBE was added and the mixture was extracted by ultrasound for 20 min at 4 °C, followed by standing for 30 min at room temperature. The solution was centrifuged at 14,000 r/min for 15 min at 10 °C, and the upper organic solvent was obtained and dried under nitrogen. The residue was redissolved in 200 µL 90% isopropanol/acetonitrile solution. Ninety μL solution was centrifuged at 14,000 r/min for 15 min and then the supernatant was injected into a vial for analysis. The QC samples were prepared by mixing the aliquot of the all samples to be a pooled sample.

A Nexera LC−30A UHPLC (SHIMADZU, Kyoto, Japan) coupled with CSH C_18_ column (2.1 mm × 100 mm, 1.7 µm) (Waters, Milford, CT, USA) was used to separate the extracted lipids in yak milk. The column temperature and the flow rate were 45 °C, 300 μL/min, respectively. The mobile phase consisted of A (acetonitrile−water (6:4, *v*:*v*) with 0.1% formic acid and 0.1 mmol/L ammonium formate) and B (acetonitrile−isopropanol (1:9, *v*:*v*) with 0.1% formic acid and 0.1 mmol/L ammonium formate). The initial mobile phase was 30% B and held for 2 min and then linearly increased to 100% B in 23 min, followed by equilibration at 5% B for 10 min. The MS data were acquired using a Q−Exactive Plus mass spectrometer (Thermo Scientific, Waltham, MA, USA) in positive and negative mode, respectively. The ESI parameters were as follows: heater temp 300 °C, sheath gas flow rate 45 arb, aux gas flow rate 15 arb, sweep gas flow rate 1arb, spray voltage 3.0 KV, capillary temp 350 °C, S−Lens radio−frequency (RF) level 50%, MS scan ranges 200–1800. LipidSearch was used for peak identification, peak extraction, and identification of lipid molecules and internal standard lipid molecules. The main parameters were as follow: precursor tolerance 5 ppm, product tolerance 5 ppm, product ion threshold 5%.

### 2.5. Metabolites Extraction and MS Data of Non−Targeted Metabolomics Based on Ultra High Performance Liquid Chromatography Tandem Quadrupole Time of Flight Mass Spectrometer (UHPLC−TOF−MS)

The metabolites in yak milk were detected according to the method described in references [22,23] with some modifications. A 100 uL milk sample, the solution of L−2−chlorophenylalanine in methanol (0.3 mg/mL), the solution of lyso PC17:0 in methanol (0.1 mg/mL), and 400 uL the solution of methanol, acetonitrile, and water solution (2:2:1, *v:v:v*) were transferred into a polyethylene (PE) tube. The mixture was vortexed to remove proteins, followed by the extraction with ultrasound for 30 min, and then was allowed to stand for 10 min at −20 °C and centrifuged at 14,000 r/min for 20 min. The supernatant was dried under vacuum and then the residue was redissolved in a 100 μL solution of acetonitrile and water (1:1, *v:v*) and centrifuged at 14,000 r/min for 15 min. The supernatant was transferred into a vial for analysis. To monitor the stability and repeatability of instrument analysis, the QC samples were prepared by pooling 10 μL of each sample and analyzed together with the other samples, and they were inserted regularly and analyzed in every 5 samples.

The prepared samples were separated with a 1290 Infinity LC UHPLC (Agilent Corp., Santa Clara, CA, USA) coupled with ACQUIY UPLC BEH Amide column (2.1 mm × 100 mm, 1.7 µm) (Waters, Milford, CT, USA). The mobile phase was composed of A (25 mmol/L ammonium acetate and 25 mmol/L ammonium hydroxide in water) and B (acetonitrile). The elution program was as follows: 95% B over 0–0.5 min, 95–65% B over 0.5–7.0 min, 65–40% B over 7.0–8.0 min, holding at 40% B from 8.0–9.0 min, 40–95% B over 9.0–9.1 min, holding at 95% B for 3 min. The column temperature, flow rate, injection volume was 25 °C, 0.5 mL/min, 2 μL, respectively. The MS data were obtained with an AB Triple TOF 6600 MS system on ESI+ and ESI- mode. The ESI source conditions were as follows: gas1 60, gas2 60, curtain gas 30, source temperature 600 °C, ISVF ± 5500 V. The accumulation time for TOF MS scan was set at 0.20 s/spectra. The product ion scan was acquired using information dependent acquisition (IDA) with high sensitivity mode selected. The parameters were as follows: collision energy 35 V ± 15 eV, declustering potential 60 V (+) and −60 V (−), exclude isotopes within 4 Da, candidate ions to monitor per cycle 10.

The raw MS data were converted to MzXML files using ProteoWizard MSConvert before importing into freely available XCMS software. For peak picking, the following parameters were used: centWave m/z = 10 ppm, peakwidth = c (10, 60), prefilter = c (10, 100). For peak grouping, bw = 5, mzwid = 0.025, minfrac = 0.5 were used. Collection of Algorithms of MEtabolite pRofile Annotation (CAMERA) was used for the annotation of isotopes and adducts. In the extracted ion features, only the variables having more than 50% of the nonzero measurement values in at least one group were kept. The compound identification of metabolites was performed by comparing of the accuracy m/z value (< 10 ppm) and the MS spectra with an in-house database established with available authentic standards. The KEGG pathway enrichment analysis was performed using MBROLE version 2.0 (http://csbg.cnb.csic.es/mbrole2/, accessed on 11 April 2023), and the KEGG enrichment scatterplot was performed by OmicStudio tools (https://www.omicstudio.cn/tool, accessed on 11 April 2023). The metabolites were blasted against the online KEGG database (http://geneontology.org/, accessed on 11 April 2023) to retrieve their COs and were subsequently mapped to the pathways in KEGG. The corresponding KEGG pathways were extracted. KEGG pathway enrichment analyses were applied based on the Fisher’s exact test, considering the whole metabolites of each pathway as the background dataset.

### 2.6. Statistical Analysis

The data of fatty acids, amino acids, and their derivatives were analyzed by the non-parametric test using software SPSS 16.0 (SPSS Inc., Chicago, IL, USA), and the *p*-values were adjusted by FDR correction. Adjusted *p*−values (*q*) < 0.05 were recognized as the significant difference. The MS data from non-targeted metabolome and lipidomics were imported into R to carry out the principal component analysis (PCA). Further, orthogonal partial least squares discriminant analysis (OPLS-DA) was used to distinguish the lipids and metabolites in yak colostrum and mature milk. The significantly different lipids (SDLs) and DMs were selected with variable importance in the projection (VIP) > 1.0 and *p* < 0.05. The KEGG analysis for DMs was performed to identify those KEGG pathways with *p* < 0.05.

## 3. Results

### 3.1. Content of Fatty Acids in Yak Colostrum and Mature Milk

In total, 17 saturated fatty acid (SFAs), 9 monounsaturated fatty acid (MUFAs) and 14 PUFAs were simultaneously detected in yak colostrum and mature milk, and the absolute and relative contents of the fatty acids are shown in Table 1. The absolute content of 16 fatty acids was different in yak colostrum and mature milk, and C4:0, C18:0, *t*−C18:1n9, C18:1n9, *t*,*t*−C18:2n6, C18:2n6 (linoleic acid, LA), C18:3n6, C18:3n3 (α-linolenic acid, ALA), C20:2n6, C22:6n3 (docosahexaenoic acid, DHA), Σunsaturated fatty acids (UFAs), ΣMUFAs, ΣPUFAs, Σn−3PUFAs and Σn−6PUFAs, total fatty acids (TFAs) in yak colostrum were all higher than the value in yak mature milk (*q* < 0.05). On the other hand, the relative content of 14 fatty acids was different (*q* < 0.05), and C18:0, *t*−C18:1n9, *t*, *t*-C18:2n6, ALA, ΣUFAs and ΣPUFAs in yak colostrum were higher (*q* < 0.05). Moreover, the value of ΣPUFAs/ΣSFAs in yak colostrum was higher (*q* < 0.05), whereas the value of Σn−6/n−3PUFAs was lower (*q* < 0.05).

### 3.2. Content of Amino Acids and Their Derivatives in Yak Colostrum and Mature Milk

In total, 19 kinds of amino acids and 6 derivatives were synchronously detected in yak colostrum and mature milk, and their content is shown in Table 2. Besides the glycine, aspartic acid, proline acid, alanine, cystine, ornithine, spermidine and tyrosine, the content of other amino acids and their derivatives was all significantly different between yak colostrum and mature milk. Of them, the content of 15 amino acids and their derivatives was higher in yak colostrum (*q* < 0.05), whereas only the content of aminoadipic acid and hydroxyproline was lower (*q* < 0.05). Especially, these amino acids affecting the function of immune system, such as tryptophan, phenylalanine, lysine, and their derivatives, such as taurine and choline, which are involved in the development of cerebral nerves, were higher in yak colostrum (*q* < 0.05). Further, the content of the total amino acids (TAAs) in yak colostrum was higher (*q* < 0.05). What is more important was that the content of essential amino acids (EAAs) and the value of EAAs/NEAAs in yak colostrum were higher too (*q* < 0.05).

### 3.3. Lipidomics in Yak Colostrum and Mature Milk

**Figure 1:**
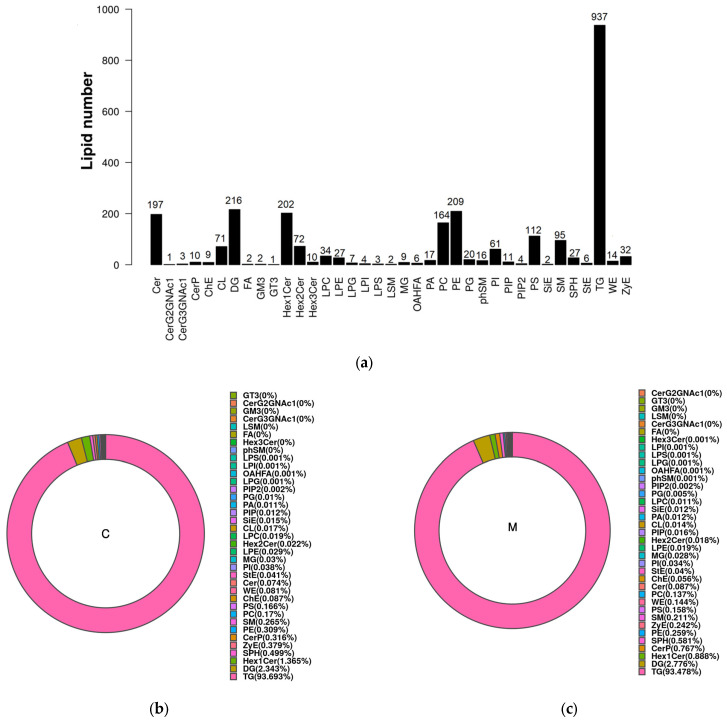
(**a**) Bar chart of the lipid subclasses and molecule numbers in yak milk. The abscissa represents the detected lipid subclass, and the ordinate represents the number of lipid molecules. (**b**) Composition of lipid subclasses in yak colostrum. C: colostrum. Different lipid subclasses are represented by the different colors, and the proportion is represented by the area size. (**c**) Composition of the lipid subclasses in yak mature milk. M: mature milk.

A total of 2615 lipid species in 37 lipid class were detected in yak milk. The numbers of lipid subclass are shown in Figure 1a. These lipids included 937 triacylglycerols (TGs), 209 phosphatidylethanolamines (PEs), 164 phosphatidylcholines (PCs), 197 ceramides (Cers), 216 diglycerides (DGs), 202 hexosyl 1 ceramides (Hex1Cers), 112 phosphatidylserines (PSs), 95 sphingomyelins (SMs), 72 hexosyl 2 ceramides (Hex2Cers), 71 cardiolipins (CLs) and 61 phosphatidylinositols (PIs). The composition of lipid subclass in yak colostrum and mature milk was shown in Figure 1b,c, respectively. The predominant lipid subclasses in both yak colostrum and mature milk were TGs (over 90%), DGs (over 2%) and Hex1Cers (over 0.8%).

**Figure 2:**
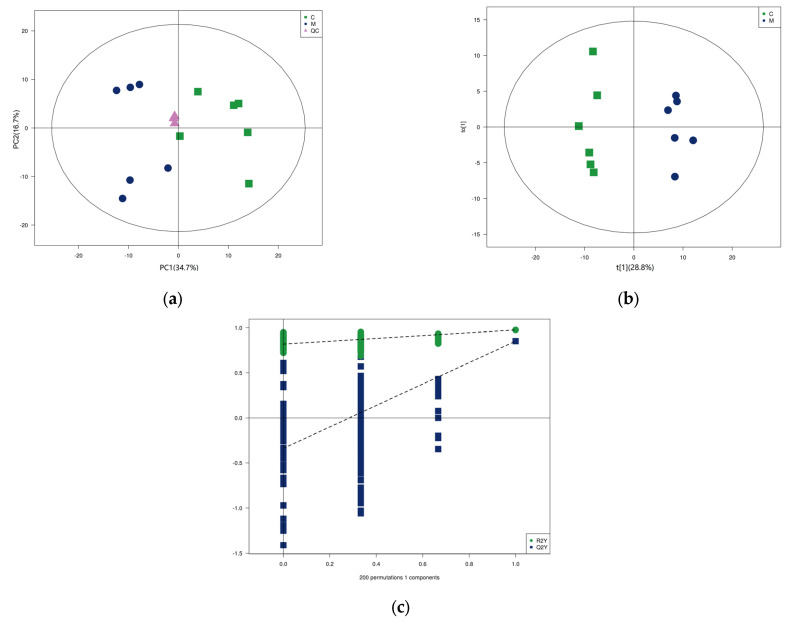
(**a**) The score plots of the principal component analysis (PCA) of lipids in yak colostrum, mature milk, and quality control (QC) samples. Green, blue, and red represent the samples of yak colostrum, mature milk, and QC, respectively. (**b**) The score plots of the orthogonal partial least squares discriminant analysis (OPLS−DA) of lipids in yak colostrum and mature milk. Green and blue represent the samples of yak colostrum and mature milk, respectively. (**c**) Permutation test of OPLS−DA model.

**Figure 3:**
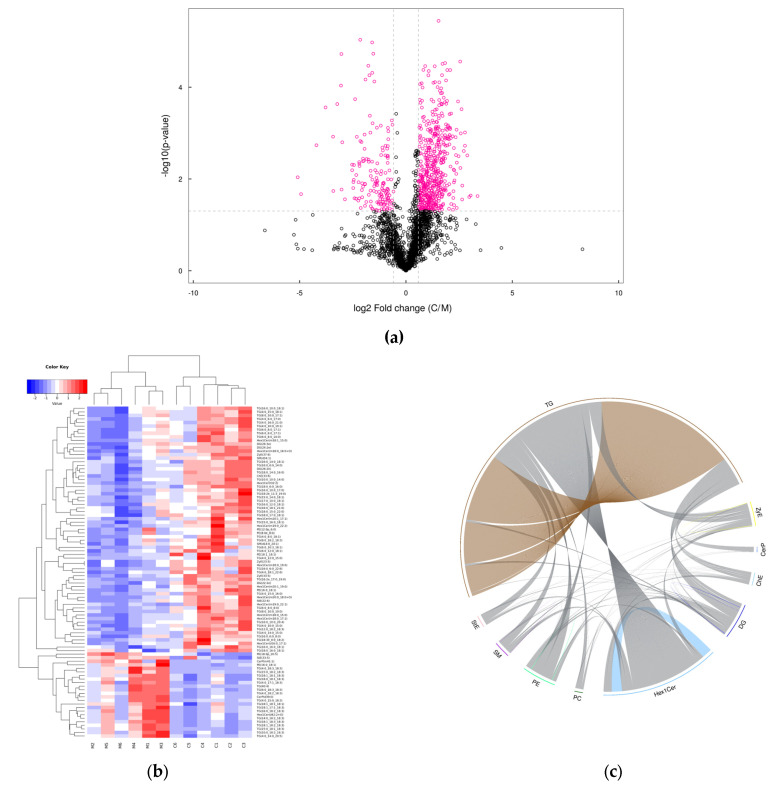
(**a**) Volcano plot of the lipids abundance in yak colostrum by contrast with mature milk. The abscissa represent the value of log_2_ fold change (FC), and the ordinate represent the value of −log_10_ *p*. A point represents a lipid molecule. The points with FC > 1.5 or < 0.67, *p* < 0.05 are shown with red, and non-significantly different lipids (SDLs) are shown with black. (**b**) Cluster heat map of SDLs. Each row represents a SDL, and each column represents a samples. The color blocks at different positions represent the abundance of lipid molecules. Red represented a high abundance, and blue represented a low abundance. (**c**) Chord diagram of lipid subclasses. The starting point of link in the inner circle represented the SDL, and the arc on the outer circle represents the lipid subclasses. The colored lines indicate the correlations of lipid molecules within the subclass, and the lines are the same color as the subclass. The dark gray lines indicate the correlations between the subclasses.

The score plots of PCA (Figure 2a) showed the distribution among yak colostrum, mature milk, and QC samples. The QC samples were congregated tightly in a small area, which indicated that the instrument was stable, and the analysis was reliable. Further OPLS−DA analysis (Figure 2b) showed that the evaluation parameters (R^2^Y, Q^2^) of the module by 7−fold cross−validation were (0.976, 0.851). Moreover, the permutation test using 200 random was used to validate the OPLS−DA models (Figure 2c). The values of R^2^Y and vertical intercept were 0.818 and −0.3382, respectively. It was found that the model was reliable, effective, and stable, and there were distinct differences in the lipids between yak colostrum and mature milk. By contrast with mature milk, the abundance change of lipid moleculars in yak colostrum is shown in Figure 3a. Total 93 SDLs were obtained and contained 2 PEs, 13 Hex1Cers, 1 PC, 3 PEs, 2 SMs, 2 StEs, 60 TGs, 4 DGs, 2 CerPs, 3 ZyEs and 1 ChE. Of these, the abundance of 69 SDLs in yak colostrum was higher (*p* < 0.05), whereas the abundance of 24 SDLs was lower (*p* < 0.05). The cluster heat map of SDLs is shown in Figure 3b. The top 10 SDLs with up−regulated fold change (FC) were TG(6:0/8:0/8:0), TG(4:0/12:0/15:0), Hex1Cer(19:0/22:2), TG(8:0/10:0/10:0), Hex1Cer(d16:0/17:1), Hex1Cer(20:0/18:0 + O), Hex1Cer(18:1/15:0), Hex1Cer(20:1/17:1), TG(18:0/6:0/16:0) and Hex1Cer(m18:0/15:0); the top 10 SDLs with down−regulated FC were TG(18:1/18:2/18:3), TG(4:0/18:3/18:3), TG(15:0/18:2/18:3), TG(40:4), TG(18:1/18:3/18:3), CerP(m41:1), TG(15:0/18:1/18:3), TG(4:0/18:2/18:3), PE(16:0/18:1) and TG(6:0/18:3/18:3). In order to more intuitively reveal the co-regulation relationship of SDLs, the chord diagram of the lipid subclasses was shown in Figure 3c. The lipid subclasses with different abundance in yak colostrum and mature milk were CerG3GNAc1, CerP, ChE, FA, GM3, GT3, Hex1Cer, LPC, LPG, LPI, PC, PG, phSM, PS, SM and ZyE. Compared with yak mature milk, the abundance of 14 lipid classes in yak colostrum was up-regulated, whereas the abundance of 2 lipid subclasses (CerP and phSM) in yak colostrum was down-regulated.

### 3.4. Metabolome in Yak Colostrum and Mature Milk

**Figure 4:**
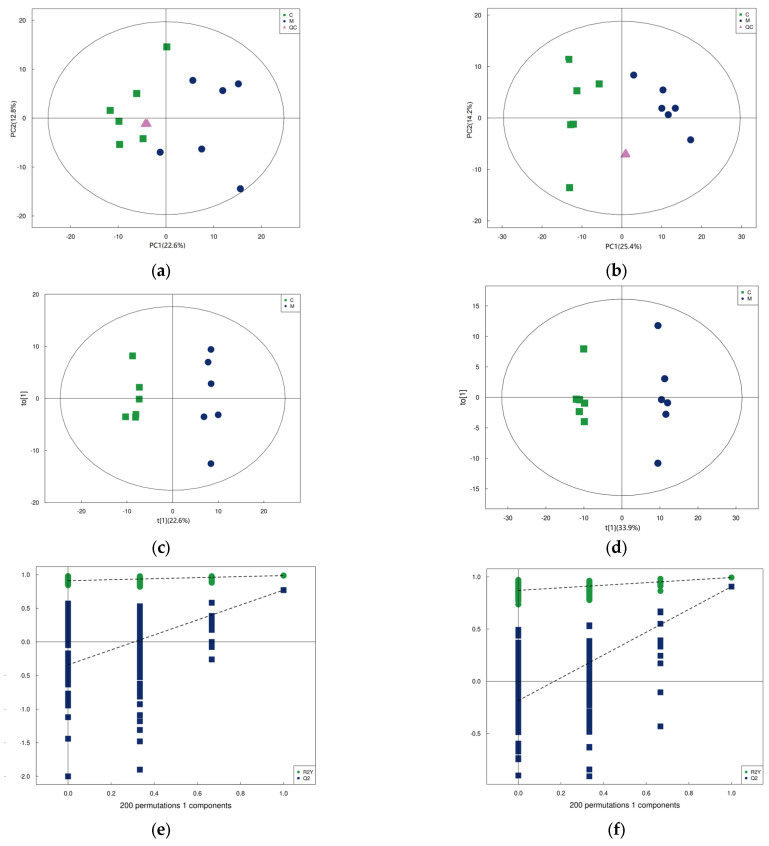
(**a**) PCA score plots of the metabolites in yak colostrum milk, mature milk, QC in positive ionization mode. Green, blue, and red represent the samples of yak colostrum milk, mature milk, QC, respectively. (**b**) PCA score plots of the metabolites in the yak colostrum milk, mature milk, QC in negative ionization mode. (**c**) OPLS−DA score plots of the metabolites in the yak colostrum and mature milk in positive ionization mode. Green and blue represent the samples of yak colostrum milk and mature milk, respectively. (**d**) OPLS−DA score plots of the metabolites in the yak colostrum and mature milk in negative ionization mode. (**e**) Permutation test of OPLS−DA in positive ionization mode. (**f**) Permutation test of OPLS−DA in negative ionization mode.

The unsupervised multivariate analyses of metabolomics data were applied to provide an initial evaluation of metabolic perturbations that had been caused by the lactation stage of yak. The score plots of PCA for the metabolites in yak milk in positive (Figure 4a) and negative (Figure 4b) ionization mode showed the distribution between yak colostrum and mature milk in two dimensions. Further analysis using OPLS-DA in positive (Figure 4c) and negative (Figure 4d) ionization mode yielded a better understanding of the variables responsible for the differentiation between yak colostrum and mature milk. The evaluation parameters (R^2^Y, Q^2^) of module by 7−fold cross−validation in positive and negative ionization mode were (0.986 0.770) and (0.993 0.906), respectively. Moreover, the permutation test using 200 random in positive (Figure 4e) and negative (Figure 4f) ionization mode was used to validate the OPLS−DA models. The values of R^2^Y and vertical intercept in positive and negative ionization mode were 0.9094 and −0.3414, 0.8702 and −0.1848, respectively. The model was considered to be reliable, effective, and stable. There were distinct differences in metabolites between yak colostrum and mature milk, which demonstrated that the lactation stage induced the marked perturbation of the metabolites in yak milk.

**Figure 5:**
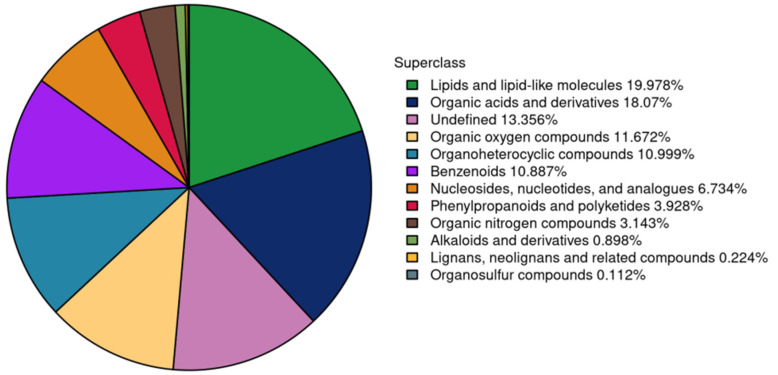
The number proportion of the identified metabolites in each chemical classification.

**Figure 6:**
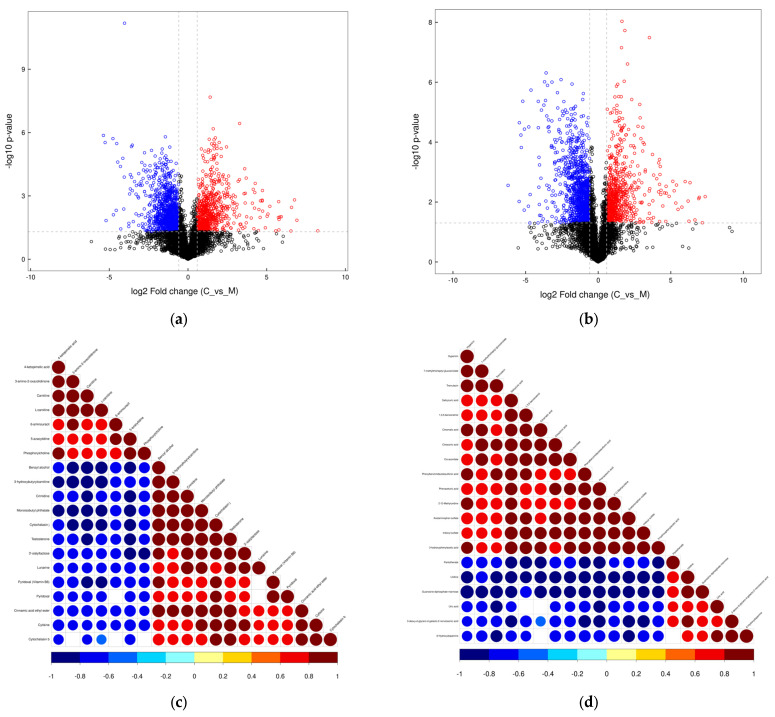
(**a**) Volcano plot of the metabolites abundance in yak colostrum by contrast with mature milk in positive ionization mode. The abscissa represents the value of log_2_ FC, and the ordinate represents the value of −log_10_ *p*. A point represents a metabolites molecule. The points with FC > 1.5 and *p* < 0.05 are shown with red; the points with FC < 0.67 and *p* < 0.05 are shown with blue; non-differential metabolites (DMs) are shown with black. (**b**) Volcano plot of the metabolites abundance in yak colostrum by contrast with mature milk in negative ionization mode. (**c**) Correlation heat map of the top 20 DMs in positive ionization mode. Red indicates the positive correlation; blue indicates the negative correlation; white indicates the non-significant correlation. The color depth was related to the absolute value of correlation coefficient. The higher the positive or negative correlation was, the darker the color was. The dot size was related to the correlation significance. The more significant the correlation was, the larger the dot was. (**d**) Correlation heat map of the top 20 DMs in negative ionization mode.

**Figure 7:**
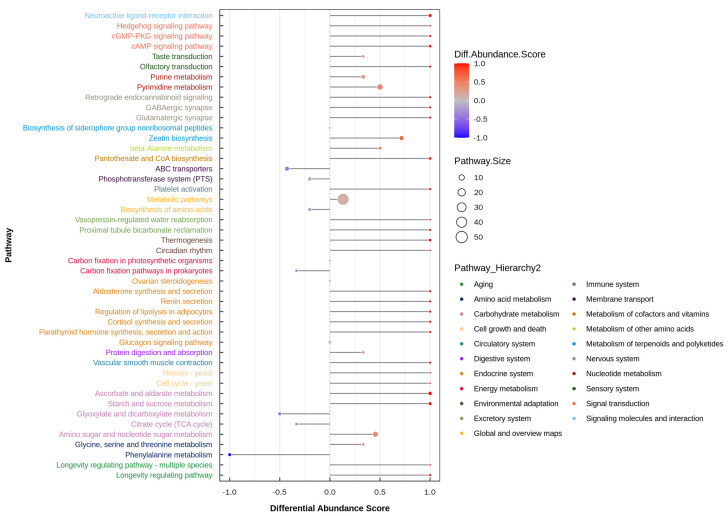
Differential abundance (DA) scores of all the enriched KEGG pathways. The *Y*-axis represented the name of metabolic pathway, and the *X*-axis represented the DA score. The DA score 1 indicated the up-regulated trend of all identified metabolites in this pathway, and −1 indicated the down-regulated trend of all identified metabolites in this pathway. The length of the line segment represented the absolute value of the DA score, and the size of the dot at the end of the line segment represented the number of metabolites in the pathway. The dot color was proportional to the DA score.

A total of 891 metabolites were detected in yak milk (Table S3), and the number proportion of the identified metabolites in each chemical classification was shown in Figure 5. In total, 503 and 388 metabolites were found in positive and negative ionization mode, respectively. The volcano plot of the metabolites abundance in yak colostrum by contrast with yak mature milk in positive and negative ionization mode is shown in Figure 6a,b, respectively. Moreover, 162 DMs in yak colostrum and mature milk were screened out, and 69 and 93 DMs were in positive and negative ionization mode (Tables S4 and S5), respectively. The abundance of 48 DMs in yak colostrum was higher, whereas the abundance of 78 DMs in yak colostrum was lower. The correlation heat map of the top 20 DMs in positive and negative ionization mode is shown in Figure 6c,d, respectively. Further, 90 DMs were enriched in 46 KEGG pathways (Table S6), and the differential abundance (DA) scores of all the enriched KEGG pathways are shown in Figure 7. These KEGG pathways are mainly involved in carbohydrate metabolism, amino acid metabolism, the endocrine system, signal transduction, the immune system, and the circulatory system.

## 4. Discussion

Cow colostrum contains abundant nutrients and a large number and variety of bioactive substances, and it possesses a high value of consumption and development [24]. The fatty acids in milk fat play an important role in the growth, development, and metabolism of newborns [25]. The predominant fatty acids in both yak colostrum and mature milk are C14:0, C16:0, C18:0, C18:1n9, *t*-C18:1n9, LA and ALA. The World Health Organization (WHO) and Food and Agriculture Organization of United Nations (FAO) suggested that the intake of ΣSFAs and trans (*t*)-fatty acids should be decreased, while the intake of ΣPUFAs, Σn-3PUFAs should be increased. The fatty acids in cow colostrum are composed of 65–75% ΣSFAs, 24–28% ΣMUFAs, and 4–5% ΣPUFAs [26]. Yak colostrum possesses a lower proportion of ΣSFAs and a higher proportion of ΣMUFAs and ΣPUFAs by contrast with cow colostrum. Meanwhile, the proportion of ΣPUFAs and Σn-3PUFAs in yak colostrum was higher than the value in yak mature milk. Therefore, it was found the fatty acids composition in yak colostrum was more reasonable for the human body by contrast with cow colostrum and yak mature milk. PUFAs can prevent diabetes and atherosclerosis and can improve cellular immunity [27], but it must be derived from precursor compounds such as linoleic acid and linolenic acid from food in the human body; accordingly, the content of PUFAs in milk is one of the crucial indexes for evaluating dairy quality. Omega-3 PUFAs can positively affect cardiovascular disease, coronary heart disease, and inflammation [28], and it can also enhance the function of the intestinal barrier and alleviate intestinal dysfunction by regulating intestinal flora. DHA and EPA can induce the development of the newborn brain [29] and also regulate the immune response of the human body by reinforcing T-lymphocyte function [30]. Moreover, the supplementation of DHA during lactation and infancy can promote the cognitive function of infants. MUFAs can reduce the blood lipids and the blood pressure of humans [31]. The content of functional fatty acids in yak mature milk was more than twice as high as the value in cow milk [7]. The content of LA, DHA, and ΣPUFAs in cow colostrum was significantly higher than the value in cow mature milk [32]. The absolute content of DHA, C22:2n6, LA, C18:3n6, ALA, ΣUFAs, ΣMUFAs, ΣPUFAs, Σn-3PUFAs and Σn-6PUFAs in yak colostrum was higher by contrast with yak mature milk. Therefore, the nutritive value of yak colostrum is higher by contrast with yak and cow mature milk from the angle of functional fatty acids. The only drawback is that yak colostrum possesses the higher content of two trans-fatty acids *t*-C18:1n9 and *t*,*t*-C18:2n6, which adversely affects the nutritional quality of yak colostrum.

The content of histidine, cysteine, TAAs, TEAAs and the value of EAAs/TAAs in yak mature milk are much higher than the values in cow and breast milk, and so yak mature milk is recommended to be used as raw of infant milk. Cow colostrum contains the higher level of serine, arginine, histidine, and taurine [33] by contrast with cow mature milk, and the same results were found in yak milk too. Further, the content of TEAAs and the value of EAAs/TAAs in yak colostrum were higher than the values in yak mature milk. Serine promotes the muscle development and the formation of cell membranes [34]; arginine maintains protein stability [35]; histidine and taurine are not only involved in signal transmission and the development of infant nervous system, but also can promote the brain development and cognitive function of infants [36]; the higher content of threonine in bovine colostrum is one of the reasons for immunomodulatory function [33]. Meanwhile, yak colostrum contained the higher content of amino acid derivative taurine and choline. Taurine plays an important role in the physiological regulation of the human body, including enhancing immunity, resisting fatigue, and maintaining retinal function [37]. Choline can improve brain metabolism, maintain the structural integrity of cell membranes, and promote the nervous system [38]. Therefore, the amino acid composition in yak colostrum is more beneficial to the human body, and the nutritional value of the amino acids in yak colostrum is higher than cow milk and yak mature milk.

The lipids in colostrum are an important means of delivering some beneficial biologically active substances. TG is the main lipid component in cow milk (98%), followed by PL and DG [39], which stays the same with the predominant composition of lipids in yak colostrum and mature milk, but compared with cow milk the content of TG in yak colostrum and mature milk was lower. The excessive intake of TG can lead to hypertension and obesity [11]. The differences of lipids in yak colostrum and mature milk were mainly decided by the glyceryl phosphatide (LPC, LPG, LPI, PC, PG and PS), sphingolipid (CerP, GM3, GT3, Hex1Cer, phSM, SM), and sterol lipids (ZyE and ChE). The polar lipids in dairy, mainly including phospholipids and sphingolipids, possess better physiological effects for humans, such as preventing intestinal digestive function, cardiovascular disease, non-alcoholic fatty liver, cognitive dysfunction, and diabetes [40,41]. The content of PCs, PGs, PSs, LPCs, LPGs, LPIs, SMs, GM3s, GT3s and Hex1Cers in yak colostrum was higher than the value in yak mature milk. Moreover, glycolipids possess the biological activities such as anti-tumor, anti-inflammation, antibacterial, and immune enhancement [42]. The content of CerG3GNAc1 belonging to glycolipids in yak colostrum was higher than the value in yak mature milk. Ceramides are involved in regulating cell differentiation and proliferation and building immune systems [43]. The content of ceramides in cow milk is very low [39], whereas the Hex1Cer was one of the main lipids in yak milk, and its content in yak colostrum was much higher than the value in mature milk. Therefore, the nutritive value of yak colostrum was higher than yak mature milk from the angle of active lipids.

The formation of cow milk is an extremely complex physiological process affected by many factors and hormones play an important role in this process [44]. Various hormones can regulate the milk synthesis in the mammary glands [45]. In this study, the KEEG pathways, including parathyroid hormone synthesis, secretion, and action (ko04928), cortisol synthesis and secretion (ko04927), aldosterone synthesis and secretion (ko04925) and ovarian steroidogenesis (ko04913), are involved in the metabolism of hormones. Estrogen and progesterone can affect milk production and quality [46,47], and their synthesis can be realized by cortisol synthesis and secretion (ko04927) and ovarian steroidogenesis (ko04913). Moreover, estrogen can regulate the blood sugar through glucagon, and glucagon metabolism is related to the glucagon signaling pathway (ko04922). On the other hand, vasopressin-regulated water reabsorption (ko04962), aldosterone synthesis and secretion (ko04925), renin secretion (ko04924), and vascular smooth muscle contraction (ko04270) are the organic component of the referencesrenin-angiotensin-aldoste-rone system, which regulates water and electrolyte balance, blood volume, and vascular tone [48,49,50]. Blood volume is one of the main factors deciding the supply of nutrient substances including fatty acids, amino acids, and carbohydrates. Therefore, the effect of the lactation period on the nutritional ingredient in yak milk was realized by the ovarian steroid hormone and the referencesrenin-angiotensin-aldosterone system. It was in accord with the above inference that quite a number of the enriched KEGG pathways focused on the carbohydrate metabolism, including starch and sucrose metabolism (ko00500), amino sugar and nucleotide sugar metabolism (ko00520), the amino acid metabolism including phenylalanine metabolism (ko00360), glycine, serine, and threonine metabolism (ko00260), the biosynthesis of amino acids (ko01230), beta-alanine metabolism (ko00410), and the fat metabolism including the regulation of lipolysis in adipocytes (ko04923). The fatty acids, amino acids, and carbohydrates in yak can be transferred by the citrate cycle (ko00020), and the differences of the nutritional ingredients in yak colostrum and mature milk were formed in the end.

## 5. Conclusions

The absolute content of the ΣMUFAs, ΣPUFAs, Σn-3PUFAs and Σn-6PUFAs in yak colostrum was higher by contrast with yak mature milk, and the fatty acid composition in yak colostrum is more reasonable than cow colostrum and yak mature milk; the content of most of the amino acids and amino acid derivatives, EAAs, and the value of EAAs/TAAs in yak colostrum were higher than the values in yak mature milk. The nutritive value of yak colostrum is higher than yak and cow mature milk from the angle of functional active amino acids and fatty acids. The content of phospholipids and sphingolipids including PCs, PGs, PSs, LPCs, LPGs, LPIs, SMs, GM3s, GT3s and Hex1Cers in yak colostrum was higher than the value in yak mature milk. In a word, the nutritive value of yak colostrum is superior to yak mature milk and cow milk. Moreover, the differences of nutritive value in yak colostrum and mature milk may be mainly regulated by the ovarian hormone and referencesrenin-angiotensin-aldosterone system affecting the lipids, amino acids, and carbohydrates metabolism in yaks. Because of the multitude of its functional active ingredients, yak colostrum is a kind of valuable raw material for infant formula milk powder, but taking sustainable stock farming into account, the collection volume of yak colostrum cannot be enough.

## Figures and Tables

**Table 1:** The absolute and relative content of fatty acids in yak colostrum and mature milk.

Compound	Absolute Content (Mean ± SD, g/L)		Relative Content (Mean ± SD, %)	
	Colostrum	Mature Milk	Colostrum	Mature Milk
C4:0	0.009 ± 0.005 *	0.004 ± 0.001	0.015 ± 0.008	0.008 ± 0.001
C6:0	0.005 ± 0.001	0.004 ± 0.001	0.008 ± 0.002	0.007 ± 0.002
C8:0	0.011 ± 0.004	0.010 ± 0.003	0.02 ± 0.01	0.02 ± 0.01
C10:0	0.13 ± 0.05 *	0.53 ± 0.27	0.22 ± 0.08 *	1.04 ± 0.22
C11:0	0.003 ± 0.001 *	0.008 ± 0.003	0.005 ± 0.002 *	0.015 ± 0.005
C12:0	0.66 ± 0.16 *	1.38 ± 0.28	1.06 ± 0.24 *	2.74 ± 0.51
C13:0	0.04 ± 0.01	0.05 ± 0.01	0.07 ± 0.02	0.09 ± 0.02
C14:0	5.33 ± 0.51	5.88 ± 0.50	8.64 ± 0.65 *	11.66 ± 0.78
C14:1n5	0.16 ± 0.04	0.18 ± 0.03	0.27 ± 0.06 *	0.36 ± 0.05
C15:0	1.27 ± 0.21	1.21 ± 0.12	2.06 ± 0.33	2.39 ± 0.19
C15:1n5	0.08 ± 0.04	0.04 ± 0.01	0.13 ± 0.07	0.09 ± 0.02
C16:0	15.79 ± 2.26	13.97 ± 1.92	25.60 ± 3.45	27.73 ± 3.59
C16:1n7	1.19 ± 0.27 *	1.62 ± 0.27	1.92 ± 0.40 *	3.23 ± 0.61
C17:0	0.82 ± 0.08 *	1.00 ± 0.02	1.33 ± 0.15 *	1.98 ± 0.07
C17:1n7	0.39 ± 0.09	0.42 ± 0.09	0.63 ± 0.13	0.84 ± 0.20
C18:0	11.26 ± 1.32 *	7.05 ± 0.53	18.32 ± 2.44 *	14.00 ± 0.93
*t*−C18:1n9	4.80 ± 1.03 *	1.41 ± 0.32	7.77 ± 1.53 *	2.82 ± 0.70
C18:1n9	14.85 ±1.51 *	12.21 ± 0.36	24.08 ± 2.21	24.28 ± 1.20
*t,t*−C18:2n6	0.137 ± 0.024 *	0.036 ± 0.004	0.22 ± 0.04 *	0.07 ± 0.01
C18:2n6 (LA)	2.03 ± 0.43 *	1.32 ± 0.20	3.29 ± 0.67	2.63 ± 0.44
C18:3n6	0.013 ± 0.002 *	0.008 ± 0.002	0.021 ± 0.004	0.017 ± 0.003
C18:3n3 (ALA)	1.36 ± 0.27 *	0.67 ± 0.11	2.21 ± 0.45 *	1.34 ± 0.23
C20:0	0.43 ± 0.08	0.42 ± 0.04	0.70 ± 0.14	0.83 ± 0.06
C20:1n9	0.08 ± 0.01	0.09 ± 0.02	0.12 ± 0.02 *	0.18 ± 0.04
C20:2n6	0.024 ± 0.004 *	0.020 ± 0.003	0.039 ± 0.01	0.040 ± 0.04
C21:0	0.07 ± 0.01	0.08 ± 0.02	0.11 ± 0.03	0.16 ± 0.03
C20:3n6	0.012 ± 0.003	0.017 ± 0.003	0.02 ± 0.01 *	0.03 ± 0.01
C20:4n6 (ARA)	0.10 ± 0.03 *	0.17 ± 0.04	0.16 ± 0.05 *	0.34 ± 0.07
C20:3n3	0.02 ± 0.003	0.02 ± 0.003	0.03 ± 0.01	0.03 ± 0.01
C22:0	0.14 ± 0.03	0.15 ± 0.03	0.22 ± 0.05	0.30 ± 0.04
C20:5n3 (EPA)	0.06 ± 0.01	0.06 ± 0.01	0.09 ± 0.02	0.11 ± 0.02
C22:1n9	0.025 ± 0.005	0.023 ± 0.004	0.041 ± 0.01	0.046 ± 0.01
C22:2n6	0.003 ± 0.0004	0.002 ± 0.004	0.004 ± 0.001	0.003 ± 0.001
C23:0	0.07 ± 0.01	0.07 ± 0.01	0.11 ± 0.03	0.14 ± 0.03
C22:4n6	0.013 ± 0.002	0.013 ± 0.003	0.020 ± 0.003	0.026 ± 0.006
C22:5n6	0.002 ± 0.001	0.002 ± 0.001	0.003 ± 0.002	0.005 ± 0.002
C24:0	0.07 ± 0.01	0.07 ± 0.01	0.11 ± 0.02	0.13 ± 0.02
C22:5n3	0.16 ± 0.03	0.11 ± 0.03	0.26 ± 0.05	0.22 ± 0.06
C24:1n9	0.008 ± 0.002	0.010 ± 0.004	0.014 ± 0.003	0.021 ± 0.007
C22:6n3 (DHA)	0.04 ± 0.01 *	0.02 ± 0.01	0.06 ± 0.01	0.04 ± 0.02
ΣTFAs	61.64 ± 2.36 *	50.35 ± 1.33	-	-
ΣSFAs	36.10 ± 2.79 *	31.87 ± 2.31	58.61 ± 4.35	63.23 ± 3.36
ΣUFAs	25.53 ± 3.02 *	18.48 ± 1.34	41.40 ± 4.35	36.77 ± 3.36
ΣMUFAs	21.57 ± 2.38 *	16.01 ± 1.00	34.97 ± 3.31	31.85 ± 2.65
ΣPUFAs	3.96 ± 0.77 *	2.47 ± 0.37	6.43 ± 1.23 *	4.91 ± 0.79
Σn−3PUFAs	2.34 ± 0.49 *	1.59 ± 0.25	2.64 ± 0.49 *	1.74 ± 0.27
Σn−6PUFAs	1.63 ± 0.29 *	0.88 ± 0.12	3.79 ± 0.77	3.17 ± 0.52
Σn−6/n−3PUFAs	1.43 ± 0.11 *	1.82 ± 0.06	1.43 ± 0.11 *	1.82 ± 0.06
ΣPUFAs/ΣSFAs	0.11 ± 0.03 *	0.08 ± 0.02	0.11 ± 0.03 *	0.08 ± 0.02

*: the content of corresponding index in yak colostrum was different from the value in yak mature milk, *q* (adjusted *p*-values with FDR corrected) < 0.05; ns: no significant difference. SD: standard deviation. *t:* trans, TFAs: total fatty acids, SFAs: saturated fatty acids, UFAs: unsaturated fatty acids, MUFAs: monounsaturated fatty acids, PUFAs: polyunsaturated fatty acids, ΣMUFAs: sum of MUFAs, ΣSFAs: sum of SFAs, ΣPUFAs: sum of PUFAs, ΣUFAs: sum of UFAs, ΣTFAs: sum of all TFAs. LA: linoleic acid, ALA: α-linolenic acid, DHA: docosahexaenoic acid, ARA: arachidonic acid, EPA: eicosapentaenoic acid.

**Table 2:** The content of amino acids and their derivatives in yak colostrum and mature milk.

Compound	Colostrum (Mean ± SD)	Mature Milk (Mean ± SD)
Lysine (g/L)	8.44 ± 0.65 *	4.17 ± 0.21
Tryptophan (g/L)	1.87 ± 0.33 *	0.81 ± 0.06
Phenylalanine (g/L)	4.53 ± 0.77 *	2.27 ± 0.23
Methionine (g/L)	2.07 ± 0.29 *	1.36 ± 0.20
Threonine (g/L)	3.24 ± 0.28 *	2.29 ± 0.30
Leucine (g/L)	6.56 ± 0.67 *	5.06 ± 0.36
Isoleucine (g/L)	3.49 ± 0.55 *	2.34 ± 0.27
Valine (g/L)	3.26 ± 0.51 *	2.56 ± 0.22
Histidine (g/L)	3.00 ± 0.37 *	1.77 ± 0.17
Glutamic acid (g/L)	19.69 ± 2.33 *	11.03 ± 0.84
Glycine (g/L)	2.70 ± 0.51	2.77 ± 0.16
Aspartic acid (g/L)	4.64 ± 0.50	4.12 ± 0.34
Arginine (g/L)	2.19 ± 0.40 *	1.48 ± 0.28
Serine (g/L)	3.61 ± 0.36 *	2.50 ± 0.39
Tyrosine (g/L)	1.93 ± 0.21	1.61 ± 0.20
Proline (g/L)	2.88 ± 0.67	2.18 ± 0.26
Alanine (g/L)	2.17 ± 0.29	1.83 ± 0.45
Cysteine (g/L)	1.35 ± 0.28 *	0.61 ± 0.08
Cystine (g/L)	0.39 ± 0.09	0.31 ± 0.06
Ornithine (mg/L)	6.50 ± 0.90	5.93 ± 0.61
Taurine (mg/L)	53.77 ± 5.53 *	22.62 ± 4.10
Choline (mg/L)	766.82 ± 39.43 *	220.54 ± 20.83
Aminoadipic acid (mg/L)	2.10 ± 0.43 *	10.77 ± 1.42
Hydroxyproline (mg/L)	3.27 ± 0.71 *	8.56 ± 1.13
Spermidine (mg/L)	9.71 ± 1.47	9.72 ± 0.86
EAAs (g/L)	36.44 ± 1.38 *	22.63 ± 0.92
NEAAs (g/L)	41.56 ± 2.15 *	28.42 ± 1.11
TAAs (g/L)	78.00 ± 3.16 *	50.89 ± 2.26
EAAs/NEAAs	0.88 ± 0.04 *	0.79 ± 0.01

The content of amino acids and their derivatives in yak colostrum marked with * was significantly different from the value in yak mature milk. EAAs: essential amino acids, NEAAs: non-essential amino acids; TAAs: total amino acids.

## Data Availability

The original contributions presented in the study are included in the article/Supplementary Material; further inquiries can be directed to the corresponding authors.

## Supplementary Materials

The following supporting information can be downloaded at: https://www.mdpi.com/article/10.3390/foods12081707/s1, Table S1: The information of standard curve for calculating the content of fatty acids; Table S2: The information of standard curve for calculating the content of amino acids and their derivatives; Table S3: The information of the total of 891 metabolites were detected in yak milk; Table S4: The information of 69 DMs in positive ionization mode; Table S5: The information of 93 DMs in negative ionization mode; Table S6: The information of 46 KEGG pathways enriched by DMs.
